# The Mediation Effect of Attitude on the Association Between Knowledge and Self-Management Behaviors in Chinese Patients With Diabetes

**DOI:** 10.3389/ijph.2023.1606022

**Published:** 2023-09-13

**Authors:** Yu Zhang, Beibei Zhang, Cunchuan Chen, Xia Feng, Suhang Song, Haipeng Wang

**Affiliations:** ^1^ Center for Health Management and Policy Research, School of Public Health, Cheeloo College of Medicine, Shandong University, Jinan, China; ^2^ NHC Key Laboratory of Health Economics and Policy Research, Shandong University, Jinan, China; ^3^ Department of Clinical Laboratory, Shandong Mental Health Center, Shandong University, Jinan, Shandong, China; ^4^ Department of Health Policy and Management, College of Public Health, University of Georgia, Athens, GA, United States

**Keywords:** diabetes, self-management, knowledge, attitudes, behaviors

## Abstract

**Objective:** This study aims to investigate the level of knowledge, attitude and self-management behaviors of diabetic patients, to explore the mediating role of attitude in the relationship between knowledge and self-management behaviors.

**Methods:** 900 diabetes patients were randomly selected from the eastern, central and western regions of Shandong Province, China, and recovered 863 valid questionnaires. Path analysis was used to examine the mediating role of attitude in the relationship between knowledge and self-management behaviors in patients with diabetes.

**Results:** The mean score (SD) of the diabetes self-management questionnaire (DSMQ) in this study was 35.01 (6.87). The direct effect value of knowledge level on self-management behaviors was 0.357, accounting for 62.09% of the total effect. The indirect effect value of knowledge on self-management behaviors through attitude was 0.218, accounting for 37.91%.

**Conclusion:** Level of knowledge has a significant direct impact on self-management behaviors and an indirect impact on self-management behaviors through attitude. Therefore, it is necessary to strengthen health education on diabetes to improve awareness, ameliorate attitudes toward diabetes, and change self-management behaviors.

## Introduction

With the rapid development of social economy and the aggravation of population aging, the prevalence of diabetes is gradually increasing [1]. According to the 10th edition of the Diabetes Atlas released by the International Diabetes Federation [2], the number of patients with diabetes aged 20–79 in the world has reached 536.6 million in 2021, and is estimated to reach 783.2 million by 2045. China is one of the countries with the most significant increase in the prevalence of diabetes in the world [3]. The number of patients with diabetes in China was about 140.9 million in 2021, ranking the first in the world, and is estimated to reach 174.4 million by 2045 [2]. Diabetes is one of the main drivers of global mortality, about 6.7 million adults aged 20–79 will die of diabetes or its complications in 2021, and China has the highest number of deaths from diabetes, at approximately 1.4 million [2]. Unstable blood glucose control may cause a series of serious complications, such as retinopathy, nephropathy, and neuropathy [4], which seriously threat the quality of life in patients with diabetes and bring a huge disease burden to individuals, families, and society. Therefore, it is particularly important to control the blood glucose in patients with diabetes and prevent the onset of complications.

Diabetes management is reported to help control blood glucose and effectively prevent complications [5]. In recent years, research on diabetes management focuses on community management and self-management [6]. Diabetes community management refers to health education, guidance, and intervention implemented for diabetic patients performed by community medical staff, and regular health monitoring and follow-up visits with the aim of treatment compliance improvement [7]. Worldwide practical experience shows that extensive community-based population intervention and health management is an effective strategy for preventing and controlling diabetes [8, 9]. In addition, a large amount of research demonstrates that active engagement in diabetes community management assists in controlling the level of blood glucose, promoting individual mental health and further improving the quality of life [10–12].

Moreover, the community management of diabetes also emphasizes the self-management of patients [13–15]. From the experience at home and abroad, the community is an important base for self-management and prevention of diabetes, and also the most effective choice to prevent and control chronic diseases. Based on community management, community health workers help improve the self-management ability of patients with diabetes through health education, and combine community management with self-management to comprehensively and systematically manage the health status. Therefore, the self-management of patients with diabetes has received more and more attention. It is considered to be one of the most cost-effective health management measures, and is described as the basis for controlling blood glucose, preventing complications, and improving health status and quality of life [15, 16]. Diabetes self-management refers to the diabetes patients, with the assistance of medical staff, managing their daily behaviors and lifestyle by adopting scientific self-management skills. It is conducive to stabilizing the condition, promoting health and improving the quality of life [15, 17].

Prior studies have found that the self-management level of patients with diabetes still needs to be improved [18, 19]. A study in Oman found that only 1% of patients regularly self-monitored their blood glucose, 9.5% of them did exercise regularly, and 18% of them maintained healthy diet habits [18]. A study from China showed that only 9.2% of patients with diabetes had a good level of self-management [19]. Numerous studies have explored the factors associated with self-management. A systematic review has reported that longer time living with diabetes, financial difficulties, and low self-efficacy were the main obstacles to diabetes self-management [20]. Another study found that males, being old, married, duration of diabetes, the COVID-19 pandemic, reduced support from health professionals, and increased levels of anxiety and stress were associated with the self-management level of patients with diabetes [21].

Previous studies on diabetes self-management mainly focus on the level of self-management [18, 19], influencing factors [22, 23], and the effect of intervention strategies [24]. Some studies have also discussed the impact of the knowledge level or attitude on self-management behaviors in patients with diabetes [25–28]. However, little is known about the correlation and path relationship between knowledge, attitude, and self-management behaviors in patients with diabetes. Reliable evidence is warranted to provide further insight for decision-makers to evaluate management strategies designed for the prevention and treatment of diabetes. Knowledge, Attitude, and Practice (KAP) model was first proposed by Dr. Gcust in the 1960s and has been widely used in the field of public health [29]. KAP model suggests that knowledge is the basis of behaviors change, attitude is the driving force for behaviors change [30]. This study aims to investigate the level of knowledge, attitude and self-management behaviors in patients with diabetes based on KAP model. The relationship between knowledge, attitude and self-management behavior of diabetes patients was explored through path analysis.

## Methods

### Study Design and Setting

This cross-sectional study was conducted in Shandong Province from August to September 2019. Shandong is a major province in east China with a vast area and large population [31]. This study used a multi-stage stratified sampling method. First, three prefectures (Qingdao, Weifang, and Liaocheng) were selected based on their geographical location and socio-economic status. Second, one urban district and one rural county were selected in each prefecture. Third, three streets and three townships were selected in each district and each county, respectively. Fourth, three communities and three villages were selected in each street and each township, respectively. Finally, a total of 27 urban community health stations (CHSs) and 27 rural village clinics (VCs) were selected as sample areas.

### Study Sample and Data Collection

This study is a cross-sectional study on knowledge, attitude and self-management behaviors of patients with diabetes. All the tests were double-tailed, α Value is 0.05, and the allowable error value is 0.05. The sample size calculated according to the formula “ $N=\frac{Z_{\alpha /2}^{2}\left(1-p\right)p}{\delta^{2}}$ ” should be 385 cases [32]. Considering the 20% loss rate, at least 482 patients with diabetes need to be included in this study.

A total of 900 diabetic patients who were included in the basic public health services, which is a long-term strategic deployment to address China’s public health problems and improve the health level of residents [33], were randomly selected as the participants from the sample areas. The inclusion criteria of diabetic patients were diagnosis of diabetes mellitus by the clinician. Diabetes patients with mental illness, communication difficulties and cognitive impairment were excluded. This study has been approved by the ethics committee of the School of Healthcare Management, Shandong University, China (ID: ECSHCMSDU20170401). All participants were voluntary to join the survey and provided written informed consent at the beginning of the study.

In this study, a questionnaire survey was used to collect data. A total of 863 valid questionnaires were collected from August to September 2019 with a response rate of 95.89% (863/900). Participants were convened in community health service stations or village clinics for one-on-one questioning by trained investigators. All questions in the questionnaire are filled out by the investigators, and both questioning and questionnaire filling were conducted simultaneously.

### Measures and Variables

The diabetes self-management questionnaire (DSMQ) was used to measure the self-management level of the participants [34]. DSMQ is a reliable and effective instrument to effectively evaluate self-management behaviors associated with blood glucose control. This study used a self-designed scale based on the diabetes prevention and treatment guidelines as well as the existing literature to measure the knowledge and attitude among patients with diabetes mellitus [35, 36]. There were 16 items in the knowledge scale of diabetic patients, with 1 point for a correct answer, and the total score ranged from 0 to 16. The diabetic attitude scale had 11 five-point Likert scale questions with options including very agreed, agreed, moderate, disagreed, very disagreed, and was successively assigned points of 1, 2, 3, 4, and 5. For items 1, 2, 3, and 11, the points were assigned on a reverse scale. The total score was calculated as the sum of the points in 11 items, ranging from 0 to 55. Less than 34 refers to a poor attitude, 34–46 refers to a moderate attitude, and higher than 46 refers to a good attitude.

In addition, we also collected some risk factors that contribute to the outcome variables through the questionnaire survey. Social-demographic variables included age (years), gender (male, female), residence area (rural, unban), marital status (unmarried, married), and educational level (illiteracy, primary school, junior school, high school or above). Diabetes-related variables included family medical history (yes, no), diabetes complication (yes, no), blood glucose level (normal, abnormal), and duration of diabetes (years). The duration of diabetes refers to years from diagnosis of diabetes to investigation.

### Statistical Analysis

IBM SPSS Statistics 25.0 was used to analyze the data. We used frequency (N), percentage (%), and mean (standard deviation [SD]) to show the socio-demographic and diabetes-related characteristics of the participants. One-way ANOVA and linear correlation analysis were used to analyze the categorical and continuous factors associated with self-management behaviors, respectively. Multiple linear regression analysis was carried out on the factors associated with self-management behaviors, and dummy variables were set for the categorical variables with more than two groups, including educational level. Path analysis was used to examine the mediation effect of attitude on the relationships between knowledge and self-management behaviors. All the tests were double-tailed and a *p* values ≤ 0.05 was considered to be statistically significant.

## Results

A total of 863 patients with diabetes (male 33.2% and female 65.8%) were enrolled in this study. The mean age (SD) was 51.21 (8.53) years, and the mean duration of diabetes (SD) was 8.69 (6.24) years. The mean score (SD) of diabetes knowledge was 11.32 (3.28), and the mean score (SD) of diabetes attitude was 44.48 (6.17). Diabetes patients from rural areas accounted for 45% (388/863), 10.3% of the patients were unmarried, only 16.2% had a high school education or above, nearly 1/3 of the patients had a family history of diabetes, 36.1% had diabetic complications, and 34.3% of the patients had abnormal blood glucose levels. Better diabetes self-management behavior was more likely to be observed in participants living in urban areas (F = 13.678, *p* < 0.001), with no diabetes complication (F = 6.914, *p* < 0.001), higher diabetes knowledge level (*r* = 0.242, *p* < 0.001) and higher diabetes attitude (*r* = 0.464, *p* < 0.001) (Table 1).

**TABLE 1:** Characteristics and the single analysis of diabetes self-management behaviors (China, 2019).

Variate	Mean ± SD/n (%)	Diabetes self-management behaviors		
		Mean	SD	F/r
All	863 (100.0)	35.01	6.87	
Gender				0.01
Male	286 (33.2)	35.04	7.53	
Female	576 (65.8)	35.01	6.53	
Residence area				13.68***
Rural	388 (45.0)	34.06	7.28	
Urban	475 (55.0)	35.79	6.42	
Marital status				3.03
Unmarried	89 (10.3)	33.82	6.48	
Married	772 (89.7)	35.16	6.91	
Educational level				2.13
Illiteracy	217 (25.2)	34.36	6.52	
Primary school	278 (32.3)	34.75	6.85	
Junior school	227 (26.3)	35.34	6.93	
High school or above	140 (16.2)	36.09	7.21	
Family medical history				0.19
Yes	285 (33.1)	35.17	6.64	
No	576 (66.9)	34.95	6.96	
Diabetes complication				6.91**
No	546 (63.9)	35.47	6.92	
Yes	309 (36.1)	34.19	6.73	
Blood glucose level				0.12
Normal	294 (34.3)	35.17	6.67	
Abnormal	564 (65.7)	34.99	6.86	
Age (years)	55.21 ± 8.53	35.01	6.87	−0.05
Duration of diabetes (years)	8.69 ± 6.24	35.01	6.87	0.04
Diabetes knowledge	11.32 ± 3.28	35.01	6.87	0.24***
Diabetes attitude	44.48 ± 6.17	35.01	6.87	0.46***

Abbreviation: SD, standard deviation.

Notes: *p < 0.05. **p < 0.01. ***p < 0.001. “F” refers to the variance value in one-way ANOVA. “r” refers to the correlation coefficient value in linear correlation analysis. The last four independent variables report “r”, and the remaining independent variables report “F”.

Table 2 presents the factors associated with diabetes self-management behaviors. In Model 1, after adjusting for the covariates, higher diabetes knowledge level (*β* = 0.272, *p* < 0.001) was associated with better diabetes self-management behaviors. In Model 2, after additionally adjusting for diabetes attitude, both diabetes knowledge (*β* = 0.174, *p* < 0.001) and diabetes attitude (*β* = 0.428, *p* < 0.001) were positively associated with diabetes self-management. However, the partial regression coefficients of diabetes knowledge decreased, indicating a mediation effect of diabetes attitude on the association between diabetes knowledge and self-management behaviors. Being younger (*β* = −0.071, *p* < 0.05), living in urban areas (*β* = 0.091, *p* < 0.01) and having less diabetes complication (*β* = −0.077, *p* < 0.05) were independently associated with greater self-management behaviors.

**TABLE 2:** Factors associated with self-management behaviors in diabetes patients by linear regression [*β* (95% CI)] (China, 2019).

	Model 1	Model 2
Gender (Ref. = Male)		
Female	0.015 (−0.786, 1.226)	−0.016 (−1.155, 0.690)
Residence area (Ref. = Rural)		
Urban	0.079 (0.100, 2.032)*	0.091 (0.361, 2.125)**
Marital status (Ref. = Unmarried)		
Married	0.044 (−0.520, 2.443)	−0.001 (−1.372, 1.326)
Educational level (Ref. = Illiteracy)		
Primary school	−0.054 (−1.999, 0.442)	−0.068 (−2.097, 0.137)
Junior school	−0.018 (−1.679, 1.127)	−0.045 (−1.970, 0.596)
High school or above	−0.002 (−1.684, 1.612)	0.021 (−1.115, 1.900)
Family medical history (Ref. = Yes)		
No	−0.022 (−1.290, 0.649)	−0.028 (−1.290, 0.477)
Diabetes complication (Ref. = No)		
Yes	−0.121 (−2.648, −0.746)***	−0.077 (−1.954, −0.208)*
Blood glucose level (Ref. = Normal)		
Abnormal	−0.054 (−1.722, 0.195)	−0.018 (−1.132, 0.624)
Age (years)	−0.104 (−0.141, −0.023)**	−0.071 (−0.109, −0.002)*
Duration of diabetes (years)	−0.004 (−0.083, 0.073)	−0.040 (−0.115, 0.029)
Diabetes knowledge	0.272 (0.409, 0.715)***	0.174 (0.214, 0.500)***
Diabetes attitude	—	0.428 (0.404, 0.543)***
Constant	33.395 (29.624, 37.165)***	14.153 (9.697, 18.610)***
*R* ^2^	0.10	0.27

Abbreviation: Ref., reference. Notes: *p < 0.05. **p < 0.01. ***p < 0.001.

Notes: Model 1 adjusted for all covariates associated with diabetes self-management behaviors without “Diabetes attitude.” Model 2 additionally included “Diabetes attitude.”

**FIGURE 1:**
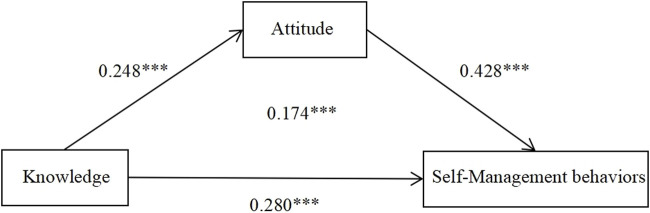
Mediation model with standardized path (*β*) coefficients of attitude as the mediator in the relationship between knowledge and self-management behaviors in patients with diabetes (China, 2019).

We further examined the mediation effect of attitude between their knowledge and self-management behaviors in patients with diabetes in Table 3. The findings indicate a partially mediation effect of attitude, since 95% CI in both direct effect (0.214, 0.500) and indirect effect (0.142, 0.299) were above 0. In addition, the direct effect of knowledge on self-management behaviors was 0.357, accounting for 62.09% of the total effect. The indirect effect of knowledge on self-management behaviors through attitude was 0.218, accounting for 37.91% (Table 3). The standardized path coefficients were shown in Figure 1.

**TABLE 3:** Mediation effect of attitude between knowledge and self-management behaviors in diabetes patients (China, 2019).

	Effect	SE	t	*p*	95% CI
Total effect of X on Y	0.575	0.079	7.320	0.000	(0.421∼0.730)
Direct effect of X on Y (c’)	0.357	0.073	4.902	0.000	(0.214∼0.500)
Indirect effect of X on Y (a × b)	0.218	0.040	—	—	(0.142∼0.299)

## Discussion

This study described the status of knowledge, attitude, and behaviors of self-management, and innovatively revealed their interrelationships in 863 Chinese patients with diabetes. Our study reported a moderate level of self-management behaviors in patients with diabetes. The level of diabetes knowledge has a significant direct impact on their self-management behaviors, and the attitude exerted a mediation effect on the association between diabetes knowledge and self-management behaviors. Findings from this study contributed to doctors understand the relationship between knowledge, attitude and self-management behaviors of diabetes patients. And these findings provide a basis for improving the self-management behaviors of diabetes patients.

The mean score of the diabetes self-management questionnaire (DSMQ) in this study was 35.01 out of 48, which was higher than that of some other countries such as Pakistan (23.04) and Iran (33.1) [37, 38], and was ameliorated compared with previous studies (30.89) in China [39], but the self-management level still lower than that of some developed countries, such as such as Germany (37.44) and Hungary (36.72) [40, 41]. The self-management level of diabetes patients varies in different studies. This may result from the differences in the socio-economic status, the access to medical and health services and diabetes self-management education in different countries and regions [37–39]. It may also be related to the different definitions of self-management behaviors in patients with diabetes, the study design and the subject factors in different studies [40–42]. Our study reported that t the self-management of diabetic patients in China is at a moderate level. Good self-management of diabetes can reduce or delay the occurrence of complications, which is conducive to the control of diabetes [43]. It is urgent to design and implement tailored approaches to improving the self-management behaviors in diabetic patients.

Our study observed that the knowledge level was positively associated with their self-management behaviors in patients with diabetes. This is consistent with the results of previous studies [44, 45]. The higher the knowledge level of diabetes patients, the better their self-management behaviors. This may be because the higher the level of diabetes knowledge of patients, the easier it is to accept opinions about changing self-management behaviors [43]. Some studies have shown that health education on diabetes has a direct impact on self-management in patients [17]. Strengthening the health education of diabetes self-management and improving the knowledge level of patients is one effective measure to improve the self-management behaviors of diabetes patients [46]. Therefore, healthcare providers should carry out health education for patients with diabetes, and actively teach patients self-management knowledge such as eating habits and physical exercise [47]. This is beneficial to change the poor self-management behavior of diabetes patients, develop good living habits, and improve the condition of diabetes.

Our study also found that attitudes toward diabetes were partially mediating the association between knowledge and self-management behaviors. It indicated that the impact of diabetes patients’ knowledge on their self-management behaviors was partly through attitude. In other words, diabetes patients with a higher level of knowledge on health were easier to form a more positive health attitude, which was conducive to developing healthy diabetes self-management behaviors. Studies have reported that a positive attitude is the basis for improving self-management behaviors in patients with diabetes [48]. Therefore, diabetes patients not only need to improve their knowledge level, but also should maintain a positive attitude, which will help improve their self-management behaviors. Some studies have shown that support received from family and friends can help diabetes patients to have an optimistic and positive attitude [49]. Therefore, the future interventions to promote good self-management behaviors among patients should involve the companionship and support of family and friends. In addition, some studies also found that patients’ attitudes towards diabetes were affected by their knowledge level [45]. Thus, in the future diabetes health education, healthcare providers should also pay attention to patients’ attitudes towards diabetes. Enhance patients’ awareness of the importance of self-management, promote patients’ positive attitude towards diabetes, and then have good self-management behaviors.

In addition, this study demonstrated significant associations between some socio-demographic characteristics and diabetes self-management behaviors. We observed that the level of self-management was better in diabetic patients living in urban areas compared to those living in rural areas. This may result from the fact that urban areas have more opportunities to access information about diabetes self-management through media, books, and the internet [50]. We also found that older patients had lower levels of diabetes self-management. The self-management level of patients with longer diabetes duration was poor. And the diabetes patients with severe complications had worse self-management behaviors. Therefore, healthcare providers should pay more attention to the above types of diabetes patients, and prevent them from worsening their diabetes due to poor self-management.

### Limitations

This study is subject to several limitations. First, this is a cross-sectional study, which can most appropriately reveal the associations of the variables observed at a single time point, instead of causality. Therefore, longitudinal datasets are warranted to identify the causality relationships in future research. Second, the knowledge and attitude scales of diabetic patients used in this study were self-designed according to previous studies, but they were proved to have acceptable Cronbach’s Alpha (0.781 and 0.701). Third, the investigators collected clinical covariates such as diabetes complication, blood glucose level, and duration of diabetes through questionnaires, which may lead to recall bias. Finally, the samples in this study were only from three prefectures in Shandong Province, which may affect the representativeness of the research samples. However, we have obtained samples from different urban districts and rural counties according to geographical location and economic development level with similar demographic characteristics to the national average, so our results still provide insights into self-management in Chinese patients with diabetes.

### Conclusion

The diabetes knowledge and attitude were positively correlated with self-management behaviors in patients with diabetes. Good self-management behaviors were based on the good knowledge level and positive attitude of patients with diabetes. The attitude toward diabetes plays a partial mediation role in the association between knowledge and self-management behaviors. These findings provide practical evidence and policy implications for diabetes-related healthcare services. Therefore, it is warranted to tailor health education programs for patients with diabetes to improve health awareness, attitudes, and self-management behaviors, and control blood glucose levels throughout all stages of diabetes.

## Data Availability

The data used and analyzed during the study are available from the corresponding author on reasonable request.
